# Bis(2-fluoro­benzoato-κ*O*)bis­(pyridin-2-amine-κ*N* ^1^)zinc(II)

**DOI:** 10.1107/S1600536809027779

**Published:** 2009-07-18

**Authors:** Jian-Quan Wang, Ya-Wen Zhang, Lin Cheng

**Affiliations:** a School of Chemistry and Chemical Engineering, Southeast University, Nanjing 211189, People’s Republic of China

## Abstract

In the title compound, [Zn(C_7_H_4_FO_2_)_2_(C_5_H_6_N_2_)_2_] or [Zn(fa)_2_(2-pa)_2_] (Hfa is 2-fluoro­benzoic acid and 2-pa = pyridin-2-amine), the asymmetric unit contains one Zn^II^ cation, two fa ligands and two 2-pa ligands, wherein the Zn^II^ displays a distorted tetra­hedral geometry, being surrounded by two monodentate fa ligands with Zn—O distances of 1.962 (2) and 1.976 (3) Å, and by two 2-pa ligands with distances involving pyridyl N atoms of 2.069 (2) and 2.056 (2) Å. The F atoms of the fa ligands are equally disordered over two sites, *viz*. the 2- and 6-positions of fa. The mononuclear complex mol­ecules are joined by N—H⋯O and N—H⋯F hydrogen bonds into a two-dimensional layer, which is further constructed into a three-dimensional supra­molecular network by weak C—H⋯F inter­actions and effective π–π stacking [centroid-centroid separation of 3.74 (3) Å] between the inter­layer aromatic rings and adjacent heterocycles.

## Related literature

For related structures, see: Darensbourg *et al.* (2002 ▶). For crystal engineering, see: Fyfe & Stoddart (1997 ▶). 
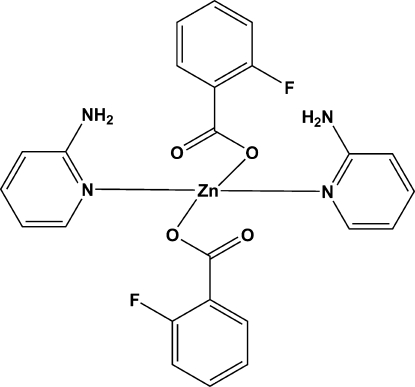


## Experimental

### 

#### Crystal data

[Zn(C_7_H_4_FO_2_)_2_(C_5_H_6_N_2_)_2_]
*M* *_r_* = 531.83
Monoclinic, 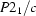

*a* = 9.1259 (7) Å
*b* = 11.1020 (9) Å
*c* = 24.5707 (17) Å
β = 108.048 (2)°
*V* = 2366.9 (3) Å^3^
*Z* = 4
Mo *K*α radiation
μ = 1.09 mm^−1^
*T* = 293 K
0.25 × 0.20 × 0.18 mm

#### Data collection

Bruker SMART APEX CCD diffractometer
Absorption correction: multi-scan (*SADABS*; Sheldrick, 2000 ▶) *T*_min_ = 0.772, *T*_max_ = 0.828
13150 measured reflections
4645 independent reflections
3838 reflections with *I* > 2σ(*I*)
*R*_int_ = 0.016

#### Refinement

*R*[*F* ^2^ > 2σ(*F* ^2^)] = 0.043
*wR*(*F* ^2^) = 0.131
*S* = 1.04
4645 reflections
334 parameters
H-atom parameters constrained
Δρ_max_ = 0.50 e Å^−3^
Δρ_min_ = −0.28 e Å^−3^

### 

Data collection: *SMART* (Bruker, 2000 ▶); cell refinement: *SAINT* (Bruker, 2000 ▶); data reduction: *SAINT*; program(s) used to solve structure: *SHELXS97* (Sheldrick, 2008 ▶); program(s) used to refine structure: *SHELXL97* (Sheldrick, 2008 ▶); molecular graphics: *SHELXTL* (Sheldrick, 2008 ▶); software used to prepare material for publication: *SHELXTL*.

## Supplementary Material

Crystal structure: contains datablocks I, global. DOI: 10.1107/S1600536809027779/pv2177sup1.cif

Structure factors: contains datablocks I. DOI: 10.1107/S1600536809027779/pv2177Isup2.hkl

Additional supplementary materials:  crystallographic information; 3D view; checkCIF report

## Figures and Tables

**Table 1:** Hydrogen-bond geometry (Å, °)

*D*—H⋯*A*	*D*—H	H⋯*A*	*D*⋯*A*	*D*—H⋯*A*
N2—H2*A*⋯O3	0.86	2.06	2.881 (3)	159
N2—H2*B*⋯O1^i^	0.86	2.03	2.864 (3)	162
N4—H4*B*⋯O2	0.86	2.02	2.838 (4)	159
N4—H4*C*⋯O4^ii^	0.86	2.03	2.884 (4)	175
N4—H4*B*⋯F2	0.86	2.52	3.143 (5)	130
C5—H5*A*⋯F4^iii^	0.93	2.34	3.147 (6)	144
C20—H20*A*⋯F3^iv^	0.93	2.47	3.244 (10)	141

Symmetry codes: (i) 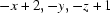; (ii) 
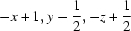
; (iii) 
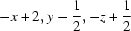
; (iv) 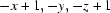.

**Fig. 1.**
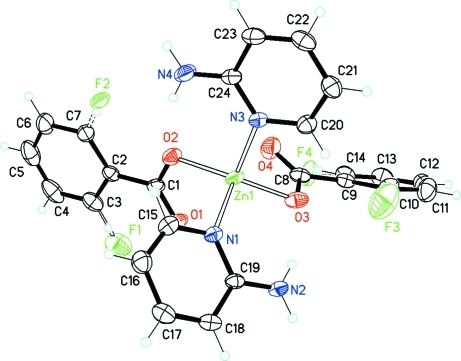
Molecular structure of the title compound with 15% thermal ellipsoids.

**Fig. 2.**
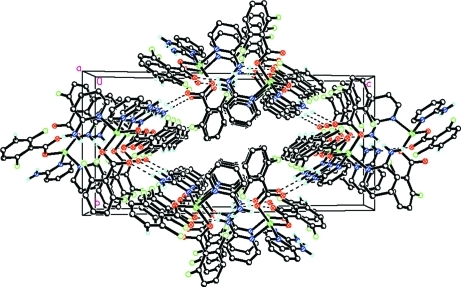
The three-dimensional supramolecular network of the title compound.

## supplementary crystallographic information

### Comment

Recently, coordination compounds of d_10_ monovalent ions of the coinage metals have attracted much attention because of their interesting photophysical properties. In order to construct many novel and interesting metal-organic frameworks carboxylates have been used widely. Moreovere, supramolecular interactions such as hydrogen bonding and π–π stacking interactions have important effects on crystal engineering (Fyfe & Stoddart, 1997). In this paper, we present the synthesis and crystal structural of a new coordination compound of d_10_ monovalent ion Zn(fa)_2_(2-pa)_2_ (Hfa = 2-fluorobenzoic acid; 2-pa = pyridin-2-amine), (I).

The asymmetric unit of the title compound, contains a Zn^II^ cation, two fa and two 2-pa ligands (Fig. 1). The Zn^II^ ion in (I) is surrounded by two monodentate fa ligands with Zn—O coordinating distances 1.962 (2) and 1.976 (3) Å and two 2-pa ligands with distances involving pyridyl N atoms and Zn being 2.069 (2) and 2.056 (2) Å; the Zn^II^ displays a distorted tetrahedral geometry with angles around Zn in the range 101.17 (10) - 137.12 (10)°. The other O-atoms of the fa ligands are at significantly longer distances from the Zn^II^ ion (Zn1—O4 2.551 (3) and Zn1—O1 (2.781 (3)Å). The mononuclear complex (I) is joined into a two-dimensional layer by N—H···O type hydrogen-bonds; details have been provided in Table 1. The layers are further constructed in to a three-dimensional supramolecular network by rather weak C—H···F type interactions (C5···F4^c^ 3.158 (2) and C20···F3^d^ 3.256 (3) Å; symmetry codes: ^c^, 1 - *x*, 1/2 + *y*, 1/2 - *z* and ^d^, -*x*, -*y*, 1 - *z*) and effective π–π stacking between the interlayer adjacent benzene rings and pyridyl rings with the centroid-centroid separation of 3.74 (3) Å. The F atoms of fa ligands in (I) are disordered over two sites, the 2- and 6-position of fa with equal site occupancy factors.

The crystal structures of a few Zn^II^ benzoate complexes have been reported by Darensbourg *et al.*, (2002).

### Experimental

A mixture of 2-fluorobenzoic acid (0.0280 g, 0.2 mmol), pyridin-2-amine(0.0188 g, 0.2 mmol), ZnSO_4_.7H_2_O (0.0285 g, 0.1 mmol) and H_2_O (8 ml) was heated in a 15-ml Teflon-lined autoclave at 393 K for 5 days, followed by slow cooling (5 ° h^-1^) to room temperature. The resulting mixture was washed with water, and colorless block crystals were collected and dried in air [yield, 62% (32.4 mg) based on Zn^II^].

### Refinement

All the H atoms were positioned geometrically and refined using a riding model with C—H = 0.93 Å and N—H = 0.86 Å with *U*_iso_(H) = 1.2*U*_iso_(C/N). The site occupancy factors of all F-atoms are given as 0.5 because of disordered position over two sites, the 2- and 6-position of fa.

### Crystal data

**Table:**

[Zn(C_7_H_4_FO_2_)_2_(C_5_H_6_N_2_)_2_]	*F*(000) = 1088
*M**_r_* = 531.83	*D*_x_ = 1.492 Mg m^−^^3^
Monoclinic, *P*2_1_/*c*	Mo *K*α radiation, λ = 0.71073 Å
Hall symbol: -P 2ybc	Cell parameters from 785 reflections
*a* = 9.1259 (7) Å	θ = 2.4–28.0°
*b* = 11.1020 (9) Å	µ = 1.09 mm^−^^1^
*c* = 24.5707 (17) Å	*T* = 293 K
β = 108.048 (2)°	Block, colorless
*V* = 2366.9 (3) Å^3^	0.25 × 0.20 × 0.18 mm
*Z* = 4	

### Data collection

**Table:**

Bruker SMART APEX CCD diffractometer	4645 independent reflections
Radiation source: fine-focus sealed tube	3838 reflections with *I* > 2σ(*I*)
graphite	*R*_int_ = 0.016
φ and ω scans	θ_max_ = 26.0°, θ_min_ = 2.0°
Absorption correction: multi-scan (*SADABS*; Sheldrick, 2000)	*h* = −10→11
*T*_min_ = 0.772, *T*_max_ = 0.828	*k* = −13→11
13150 measured reflections	*l* = −30→26

### Refinement

**Table:**

Refinement on *F*^2^	Primary atom site location: structure-invariant direct methods
Least-squares matrix: full	Secondary atom site location: difference Fourier map
*R*[*F*^2^ > 2σ(*F*^2^)] = 0.043	Hydrogen site location: inferred from neighbouring sites
*wR*(*F*^2^) = 0.131	H-atom parameters constrained
*S* = 1.04	*w* = 1/[σ^2^(*F*_o_^2^) + (0.077*P*)^2^ + 0.6445*P*] where *P* = (*F*_o_^2^ + 2*F*_c_^2^)/3
4645 reflections	(Δ/σ)_max_ = 0.001
334 parameters	Δρ_max_ = 0.50 e Å^−^^3^
0 restraints	Δρ_min_ = −0.28 e Å^−^^3^

### Special details

**Table:**

Geometry. All e.s.d.'s (except the e.s.d. in the dihedral angle between two l.s. planes) are estimated using the full covariance matrix. The cell e.s.d.'s are taken into account individually in the estimation of e.s.d.'s in distances, angles and torsion angles; correlations between e.s.d.'s in cell parameters are only used when they are defined by crystal symmetry. An approximate (isotropic) treatment of cell e.s.d.'s is used for estimating e.s.d.'s involving l.s. planes.
Refinement. Refinement of *F*^2^ against ALL reflections. The weighted *R*-factor *wR* and goodness of fit *S* are based on *F*^2^, conventional *R*-factors *R* are based on *F*, with *F* set to zero for negative *F*^2^. The threshold expression of *F*^2^ > σ(*F*^2^) is used only for calculating *R*-factors(gt) *etc*. and is not relevant to the choice of reflections for refinement. *R*-factors based on *F*^2^ are statistically about twice as large as those based on *F*, and *R*- factors based on ALL data will be even larger.

### Fractional atomic coordinates and isotropic or equivalent isotropic displacement parameters (Å^2^)

**Table:**

	*x*	*y*	*z*	*U*_iso_*/*U*_eq_	Occ. (<1)
Zn1	0.69105 (4)	−0.07190 (3)	0.378555 (11)	0.06568 (15)	
O1	0.9562 (3)	0.0314 (3)	0.36588 (9)	0.1045 (8)	
O2	0.7767 (3)	−0.0875 (2)	0.31487 (9)	0.0835 (6)	
O3	0.6376 (3)	0.06015 (18)	0.42292 (10)	0.0884 (7)	
O4	0.5834 (4)	0.1323 (3)	0.33750 (11)	0.1169 (9)	
N1	0.8367 (2)	−0.1820 (2)	0.43970 (9)	0.0634 (5)	
N2	0.8527 (3)	−0.0615 (2)	0.51811 (10)	0.0716 (6)	
H2A	0.7811	−0.0165	0.4967	0.086*	
H2B	0.8928	−0.0447	0.5538	0.086*	
N3	0.4880 (2)	−0.1672 (2)	0.35345 (9)	0.0638 (5)	
N4	0.5189 (4)	−0.2413 (3)	0.27024 (11)	0.0985 (9)	
H4B	0.6070	−0.2060	0.2786	0.118*	
H4C	0.4860	−0.2826	0.2392	0.118*	
C1	0.9007 (3)	−0.0286 (3)	0.32286 (11)	0.0658 (6)	
C2	0.9760 (3)	−0.0354 (3)	0.27689 (12)	0.0663 (7)	
C3	1.1117 (4)	0.0251 (3)	0.28201 (18)	0.0943 (10)	
H3A	1.1558	0.0718	0.3144	0.113*	0.50
F1	1.1788 (7)	0.0903 (6)	0.3380 (3)	0.155 (2)	0.50
C4	1.1843 (5)	0.0177 (5)	0.2398 (3)	0.1265 (18)	
H4A	1.2753	0.0594	0.2435	0.152*	
C5	1.1190 (7)	−0.0514 (6)	0.1936 (3)	0.136 (2)	
H5A	1.1673	−0.0575	0.1655	0.163*	
C6	0.9885 (6)	−0.1112 (5)	0.18654 (18)	0.1150 (14)	
H6A	0.9461	−0.1573	0.1538	0.138*	
C7	0.9163 (4)	−0.1044 (3)	0.22799 (13)	0.0822 (9)	
H7A	0.8255	−0.1471	0.2231	0.099*	0.50
F2	0.7836 (4)	−0.1724 (4)	0.21912 (14)	0.0941 (11)	0.50
C8	0.5867 (3)	0.1415 (3)	0.38668 (13)	0.0768 (8)	
C9	0.5172 (3)	0.2517 (3)	0.40796 (12)	0.0707 (7)	
C10	0.4409 (5)	0.2449 (4)	0.44744 (17)	0.1011 (11)	
H10A	0.4357	0.1708	0.4644	0.121*	0.50
F3	0.4654 (14)	0.1351 (9)	0.4726 (4)	0.224 (4)	0.50
C11	0.3718 (6)	0.3414 (5)	0.4633 (2)	0.1287 (17)	
H11A	0.3202	0.3320	0.4902	0.154*	
C12	0.3779 (5)	0.4498 (5)	0.4402 (2)	0.1193 (15)	
H12A	0.3288	0.5154	0.4505	0.143*	
C13	0.4567 (5)	0.4637 (4)	0.4015 (2)	0.1106 (13)	
H13A	0.4655	0.5390	0.3862	0.133*	
C14	0.5223 (4)	0.3637 (3)	0.38576 (17)	0.0946 (10)	
H14A	0.5730	0.3728	0.3585	0.114*	0.50
F4	0.6067 (6)	0.3817 (4)	0.3530 (2)	0.1135 (15)	0.50
C15	0.8819 (4)	−0.2827 (3)	0.41838 (13)	0.0803 (8)	
H15A	0.8346	−0.3002	0.3799	0.096*	
C16	0.9906 (4)	−0.3591 (3)	0.44900 (17)	0.0921 (9)	
H16A	1.0179	−0.4272	0.4323	0.111*	
C17	1.0612 (4)	−0.3324 (3)	0.50709 (16)	0.0888 (9)	
H17A	1.1377	−0.3827	0.5296	0.107*	
C18	1.0180 (3)	−0.2340 (3)	0.53020 (13)	0.0768 (8)	
H18A	1.0644	−0.2162	0.5687	0.092*	
C19	0.9016 (3)	−0.1578 (2)	0.49576 (10)	0.0606 (6)	
C20	0.4016 (4)	−0.1603 (3)	0.38926 (13)	0.0765 (7)	
H20A	0.4384	−0.1141	0.4223	0.092*	
C21	0.2633 (4)	−0.2177 (3)	0.37954 (17)	0.0867 (9)	
H21A	0.2080	−0.2120	0.4055	0.104*	
C22	0.2084 (4)	−0.2842 (3)	0.32992 (18)	0.0908 (10)	
H22A	0.1143	−0.3238	0.3218	0.109*	
C23	0.2914 (4)	−0.2918 (3)	0.29318 (14)	0.0833 (9)	
H23A	0.2541	−0.3366	0.2598	0.100*	
C24	0.4341 (3)	−0.2322 (2)	0.30510 (12)	0.0692 (7)	

### Atomic displacement parameters (Å^2^)

**Table:**

	*U*^11^	*U*^22^	*U*^33^	*U*^12^	*U*^13^	*U*^23^
Zn1	0.0722 (2)	0.0778 (2)	0.04154 (19)	−0.01443 (14)	0.00970 (14)	−0.00021 (12)
O1	0.1209 (19)	0.1082 (17)	0.0622 (13)	−0.0016 (15)	−0.0042 (12)	−0.0207 (12)
O2	0.0868 (14)	0.1085 (16)	0.0585 (11)	−0.0235 (12)	0.0275 (10)	−0.0056 (10)
O3	0.1029 (16)	0.0703 (13)	0.0712 (13)	−0.0056 (10)	−0.0036 (12)	0.0099 (9)
O4	0.143 (2)	0.129 (2)	0.0806 (17)	0.0444 (19)	0.0377 (16)	0.0251 (16)
N1	0.0669 (12)	0.0705 (13)	0.0498 (11)	−0.0109 (10)	0.0136 (9)	0.0004 (9)
N2	0.0803 (15)	0.0780 (15)	0.0471 (11)	−0.0004 (11)	0.0059 (11)	−0.0008 (10)
N3	0.0636 (12)	0.0679 (13)	0.0518 (11)	−0.0068 (10)	0.0061 (9)	0.0010 (9)
N4	0.0970 (19)	0.125 (2)	0.0697 (16)	−0.0311 (17)	0.0199 (14)	−0.0357 (16)
C1	0.0700 (16)	0.0686 (15)	0.0487 (13)	0.0060 (13)	0.0035 (11)	0.0076 (12)
C2	0.0594 (14)	0.0699 (15)	0.0645 (15)	0.0093 (12)	0.0116 (12)	0.0204 (13)
C3	0.0734 (19)	0.088 (2)	0.115 (3)	0.0021 (17)	0.0206 (19)	0.031 (2)
F1	0.119 (4)	0.137 (5)	0.195 (7)	−0.042 (3)	0.026 (4)	−0.008 (4)
C4	0.077 (2)	0.139 (4)	0.174 (5)	0.014 (3)	0.054 (3)	0.070 (4)
C5	0.120 (4)	0.180 (6)	0.133 (4)	0.058 (4)	0.074 (4)	0.066 (4)
C6	0.110 (3)	0.167 (4)	0.078 (2)	0.039 (3)	0.043 (2)	0.016 (3)
C7	0.0791 (19)	0.108 (2)	0.0595 (16)	0.0155 (17)	0.0223 (14)	0.0096 (16)
F2	0.085 (2)	0.134 (3)	0.0648 (19)	−0.030 (2)	0.0249 (17)	−0.040 (2)
C8	0.0685 (16)	0.0784 (19)	0.0685 (17)	−0.0052 (14)	−0.0005 (13)	0.0200 (15)
C9	0.0593 (14)	0.0695 (17)	0.0711 (16)	−0.0056 (12)	0.0024 (12)	0.0139 (13)
C10	0.131 (3)	0.086 (2)	0.091 (2)	−0.030 (2)	0.042 (2)	0.0014 (19)
F3	0.339 (13)	0.194 (8)	0.187 (7)	−0.059 (8)	0.150 (8)	−0.001 (6)
C11	0.136 (4)	0.135 (4)	0.136 (4)	−0.039 (3)	0.073 (3)	−0.033 (3)
C12	0.094 (3)	0.118 (4)	0.144 (4)	0.003 (2)	0.035 (3)	−0.021 (3)
C13	0.113 (3)	0.076 (2)	0.129 (3)	0.018 (2)	0.017 (3)	0.025 (2)
C14	0.089 (2)	0.089 (2)	0.102 (2)	0.0056 (18)	0.0255 (19)	0.029 (2)
F4	0.172 (4)	0.090 (3)	0.121 (3)	−0.015 (3)	0.106 (3)	0.015 (2)
C15	0.093 (2)	0.081 (2)	0.0661 (17)	−0.0059 (16)	0.0231 (15)	−0.0037 (15)
C16	0.092 (2)	0.080 (2)	0.106 (3)	0.0051 (18)	0.034 (2)	−0.0042 (19)
C17	0.0722 (18)	0.080 (2)	0.104 (2)	0.0020 (15)	0.0115 (17)	0.0141 (19)
C18	0.0678 (16)	0.0799 (19)	0.0685 (16)	−0.0092 (14)	0.0003 (13)	0.0102 (14)
C19	0.0559 (13)	0.0696 (15)	0.0516 (12)	−0.0148 (11)	0.0097 (10)	0.0071 (11)
C20	0.0832 (19)	0.0736 (18)	0.0721 (17)	−0.0086 (14)	0.0235 (15)	−0.0016 (14)
C21	0.0767 (19)	0.079 (2)	0.109 (3)	−0.0037 (16)	0.0346 (18)	0.0059 (18)
C22	0.0663 (17)	0.0722 (19)	0.122 (3)	−0.0099 (14)	0.0109 (19)	0.0084 (19)
C23	0.0765 (18)	0.0679 (17)	0.085 (2)	−0.0096 (14)	−0.0043 (16)	−0.0052 (15)
C24	0.0690 (15)	0.0647 (15)	0.0618 (15)	−0.0022 (12)	0.0025 (12)	−0.0003 (12)

### Geometric parameters (Å, °)

**Table:**

Zn1—O2	1.962 (2)	C7—F2	1.386 (5)
Zn1—O3	1.976 (3)	C7—H7A	0.9300
Zn1—N3	2.056 (2)	C8—C9	1.542 (5)
Zn1—N1	2.069 (2)	C9—C10	1.360 (5)
Zn1—O4	2.551 (3)	C9—C14	1.365 (4)
Zn1—O1	2.781 (3)	C10—F3	1.353 (10)
O1—C1	1.219 (3)	C10—C11	1.359 (6)
O2—C1	1.268 (3)	C10—H10A	0.9300
O3—C8	1.252 (3)	C11—C12	1.339 (7)
O4—C8	1.204 (4)	C11—H11A	0.9300
N1—C19	1.347 (3)	C12—C13	1.368 (7)
N1—C15	1.352 (4)	C12—H12A	0.9300
N2—C19	1.340 (4)	C13—C14	1.372 (6)
N2—H2A	0.8599	C13—H13A	0.9300
N2—H2B	0.8603	C14—F4	1.291 (5)
N3—C24	1.346 (3)	C14—H14A	0.9300
N3—C20	1.353 (4)	C15—C16	1.344 (5)
N4—C24	1.324 (4)	C15—H15A	0.9300
N4—H4B	0.8601	C16—C17	1.404 (5)
N4—H4C	0.8601	C16—H16A	0.9300
C1—C2	1.495 (4)	C17—C18	1.345 (5)
C2—C3	1.380 (4)	C17—H17A	0.9300
C2—C7	1.386 (5)	C18—C19	1.416 (4)
C3—C4	1.395 (6)	C18—H18A	0.9300
C3—F1	1.506 (8)	C20—C21	1.367 (4)
C3—H3A	0.9300	C20—H20A	0.9300
C4—C5	1.347 (8)	C21—C22	1.380 (5)
C4—H4A	0.9300	C21—H21A	0.9300
C5—C6	1.327 (8)	C22—C23	1.349 (5)
C5—H5A	0.9300	C22—H22A	0.9300
C6—C7	1.375 (5)	C23—C24	1.408 (4)
C6—H6A	0.9300	C23—H23A	0.9300
			
O2—Zn1—O3	137.12 (10)	C10—C9—C14	115.1 (3)
O2—Zn1—N3	105.09 (9)	C10—C9—C8	123.6 (3)
O3—Zn1—N3	101.17 (10)	C14—C9—C8	121.3 (3)
O2—Zn1—N1	101.66 (10)	F3—C10—C11	127.2 (5)
O3—Zn1—N1	104.50 (9)	F3—C10—C9	109.1 (5)
N3—Zn1—N1	103.33 (8)	C11—C10—C9	123.1 (4)
O2—Zn1—O4	87.86 (9)	C11—C10—H10A	118.5
O3—Zn1—O4	55.12 (9)	C9—C10—H10A	118.5
N3—Zn1—O4	97.91 (10)	C12—C11—C10	120.3 (4)
N1—Zn1—O4	153.41 (10)	C12—C11—H11A	119.9
C1—O2—Zn1	112.85 (18)	C10—C11—H11A	119.9
C8—O3—Zn1	104.1 (2)	C11—C12—C13	119.7 (5)
C19—N1—C15	118.1 (2)	C11—C12—H12A	120.2
C19—N1—Zn1	127.21 (19)	C13—C12—H12A	120.2
C15—N1—Zn1	114.31 (18)	C12—C13—C14	118.3 (4)
C19—N2—H2A	120.0	C12—C13—H13A	120.8
C19—N2—H2B	120.0	C14—C13—H13A	120.8
H2A—N2—H2B	120.0	F4—C14—C9	119.4 (4)
C24—N3—C20	118.5 (2)	F4—C14—C13	116.6 (4)
C24—N3—Zn1	126.11 (19)	C9—C14—C13	123.5 (4)
C20—N3—Zn1	115.36 (18)	C9—C14—H14A	118.2
C24—N4—H4B	120.0	C13—C14—H14A	118.2
C24—N4—H4C	120.0	C16—C15—N1	124.5 (3)
H4B—N4—H4C	120.0	C16—C15—H15A	117.8
O1—C1—O2	121.8 (3)	N1—C15—H15A	117.8
O1—C1—C2	121.2 (3)	C15—C16—C17	117.5 (3)
O2—C1—C2	117.0 (2)	C15—C16—H16A	121.2
C3—C2—C7	116.6 (3)	C17—C16—H16A	121.2
C3—C2—C1	121.1 (3)	C18—C17—C16	119.9 (3)
C7—C2—C1	122.3 (3)	C18—C17—H17A	120.1
C2—C3—C4	121.6 (4)	C16—C17—H17A	120.1
C2—C3—F1	114.6 (4)	C17—C18—C19	119.8 (3)
C4—C3—F1	123.6 (5)	C17—C18—H18A	120.1
C2—C3—H3A	119.2	C19—C18—H18A	120.1
C4—C3—H3A	119.2	N2—C19—N1	118.8 (2)
C5—C4—C3	118.2 (4)	N2—C19—C18	121.0 (2)
C5—C4—H4A	120.9	N1—C19—C18	120.2 (3)
C3—C4—H4A	120.9	N3—C20—C21	123.5 (3)
C6—C5—C4	122.7 (5)	N3—C20—H20A	118.2
C6—C5—H5A	118.7	C21—C20—H20A	118.2
C4—C5—H5A	118.7	C20—C21—C22	117.8 (3)
C5—C6—C7	119.5 (5)	C20—C21—H21A	121.1
C5—C6—H6A	120.3	C22—C21—H21A	121.1
C7—C6—H6A	120.3	C23—C22—C21	120.0 (3)
C6—C7—F2	116.7 (4)	C23—C22—H22A	120.0
C6—C7—C2	121.5 (4)	C21—C22—H22A	120.0
F2—C7—C2	121.8 (3)	C22—C23—C24	120.4 (3)
C6—C7—H7A	119.3	C22—C23—H23A	119.8
C2—C7—H7A	119.3	C24—C23—H23A	119.8
O4—C8—O3	122.8 (3)	N4—C24—N3	119.0 (3)
O4—C8—C9	121.5 (3)	N4—C24—C23	121.2 (3)
O3—C8—C9	115.6 (3)	N3—C24—C23	119.8 (3)
			
O3—Zn1—O2—C1	51.4 (3)	C3—C2—C7—F2	177.7 (3)
N3—Zn1—O2—C1	177.2 (2)	C1—C2—C7—F2	−0.7 (5)
N1—Zn1—O2—C1	−75.3 (2)	Zn1—O4—C8—O3	2.2 (3)
O4—Zn1—O2—C1	79.6 (2)	Zn1—O4—C8—C9	−173.4 (3)
O2—Zn1—O3—C8	36.5 (3)	Zn1—O3—C8—O4	−2.9 (4)
N3—Zn1—O3—C8	−90.5 (2)	Zn1—O3—C8—C9	173.00 (19)
N1—Zn1—O3—C8	162.39 (19)	O4—C8—C9—C10	142.4 (4)
O4—Zn1—O3—C8	1.38 (19)	O3—C8—C9—C10	−33.5 (4)
O2—Zn1—O4—C8	−158.3 (2)	O4—C8—C9—C14	−34.3 (5)
O3—Zn1—O4—C8	−1.4 (2)	O3—C8—C9—C14	149.8 (3)
N3—Zn1—O4—C8	96.7 (2)	C14—C9—C10—F3	−170.2 (6)
N1—Zn1—O4—C8	−46.2 (3)	C8—C9—C10—F3	12.9 (7)
O2—Zn1—N1—C19	135.2 (2)	C14—C9—C10—C11	1.1 (6)
O3—Zn1—N1—C19	−10.5 (2)	C8—C9—C10—C11	−175.8 (4)
N3—Zn1—N1—C19	−116.0 (2)	F3—C10—C11—C12	169.1 (8)
O4—Zn1—N1—C19	26.1 (3)	C9—C10—C11—C12	−0.6 (7)
O2—Zn1—N1—C15	−37.3 (2)	C10—C11—C12—C13	−1.3 (8)
O3—Zn1—N1—C15	177.0 (2)	C11—C12—C13—C14	2.6 (7)
N3—Zn1—N1—C15	71.5 (2)	C10—C9—C14—F4	172.2 (4)
O4—Zn1—N1—C15	−146.4 (2)	C8—C9—C14—F4	−10.8 (6)
O2—Zn1—N3—C24	4.1 (2)	C10—C9—C14—C13	0.2 (5)
O3—Zn1—N3—C24	149.9 (2)	C8—C9—C14—C13	177.2 (3)
N1—Zn1—N3—C24	−102.1 (2)	C12—C13—C14—F4	−174.2 (5)
O4—Zn1—N3—C24	94.0 (2)	C12—C13—C14—C9	−2.0 (6)
O2—Zn1—N3—C20	−174.6 (2)	C19—N1—C15—C16	−1.8 (4)
O3—Zn1—N3—C20	−28.8 (2)	Zn1—N1—C15—C16	171.4 (3)
N1—Zn1—N3—C20	79.2 (2)	N1—C15—C16—C17	0.2 (5)
O4—Zn1—N3—C20	−84.7 (2)	C15—C16—C17—C18	0.7 (5)
Zn1—O2—C1—O1	−0.8 (4)	C16—C17—C18—C19	−0.1 (5)
Zn1—O2—C1—C2	179.51 (18)	C15—N1—C19—N2	−176.8 (2)
O1—C1—C2—C3	0.7 (4)	Zn1—N1—C19—N2	10.9 (3)
O2—C1—C2—C3	−179.6 (3)	C15—N1—C19—C18	2.4 (4)
O1—C1—C2—C7	179.1 (3)	Zn1—N1—C19—C18	−169.80 (19)
O2—C1—C2—C7	−1.3 (4)	C17—C18—C19—N2	177.7 (3)
C7—C2—C3—C4	0.5 (5)	C17—C18—C19—N1	−1.6 (4)
C1—C2—C3—C4	178.9 (3)	C24—N3—C20—C21	1.2 (4)
C7—C2—C3—F1	−175.1 (4)	Zn1—N3—C20—C21	180.0 (3)
C1—C2—C3—F1	3.3 (5)	N3—C20—C21—C22	−1.2 (5)
C2—C3—C4—C5	−0.5 (6)	C20—C21—C22—C23	0.6 (5)
F1—C3—C4—C5	174.7 (5)	C21—C22—C23—C24	0.1 (5)
C3—C4—C5—C6	0.7 (8)	C20—N3—C24—N4	−178.8 (3)
C4—C5—C6—C7	−0.8 (8)	Zn1—N3—C24—N4	2.5 (4)
C5—C6—C7—F2	−177.7 (4)	C20—N3—C24—C23	−0.5 (4)
C5—C6—C7—C2	0.7 (6)	Zn1—N3—C24—C23	−179.2 (2)
C3—C2—C7—C6	−0.5 (5)	C22—C23—C24—N4	178.2 (3)
C1—C2—C7—C6	−178.9 (3)	C22—C23—C24—N3	−0.1 (5)

### Hydrogen-bond geometry (Å, °)

**Table:**

*D*—H···*A*	*D*—H	H···*A*	*D*···*A*	*D*—H···*A*
N2—H2A···O3	0.86	2.06	2.881 (3)	159
N2—H2B···O1^i^	0.86	2.03	2.864 (3)	162
N4—H4B···O2	0.86	2.02	2.838 (4)	159
N4—H4B···F2	0.86	2.52	3.143 (5)	130
N4—H4C···O4^ii^	0.86	2.03	2.884 (4)	175
C5—H5A···F4^iii^	0.93	2.34	3.147 (6)	144
C20—H20A···F3^iv^	0.93	2.47	3.244 (10)	141

Symmetry codes: (i) −*x*+2, −*y*, −*z*+1; (ii) −*x*+1, *y*−1/2, −*z*+1/2; (iii) −*x*+2, *y*−1/2, −*z*+1/2; (iv) −*x*+1, −*y*, −*z*+1.
